# Ca^2+^-dependent recruitment of voltage-gated sodium channels underlies bilirubin-induced overexcitation and neurotoxicity

**DOI:** 10.1038/s41419-019-1979-1

**Published:** 2019-10-10

**Authors:** Hao-Song Shi, Ke Lai, Xin-Lu Yin, Min Liang, Hai-Bo Ye, Hai-Bo Shi, Lu-Yang Wang, Shan-Kai Yin

**Affiliations:** 1 Department of Otorhinolaryngology, The Sixth People’s Hospital of Shanghai and Shanghai Jiao Tong University, 600 Yishan Road, 200233 Shanghai, P. R. China; 2 Programs in Neurosciences & Mental Health, SickKids Research Institute and Department of Physiology, University of Toronto, Toronto, ON M5G 1X8 Canada

**Keywords:** Cell death in the nervous system, Neurophysiology

## Abstract

Neonatal jaundice is prevalent among newborns and can lead to severe neurological deficits, particularly sensorimotor dysfunction. Previous studies have shown that bilirubin (BIL) enhances the intrinsic excitability of central neurons and this can potentially contribute to their overexcitation, Ca^2+^ overload, and neurotoxicity. However, the cellular mechanisms underlying elevated neuronal excitability remain unknown. By performing patch-clamp recordings from neonatal neurons in the rat medial vestibular nucleus (MVN), a crucial relay station for locomotor and balance control, we found that BIL (3 μM) drastically increases the spontaneous firing rates by upregulating the current-mediated voltage-gated sodium channels (VGSCs), while shifting their voltage-dependent activation toward more hyperpolarized potentials. Immunofluorescence labeling and western immunoblotting with an anti-NaV1.1 antibody, revealed that BIL elevates the expression of VGSCs by promoting their recruitment to the membrane. Furthermore, we found that this VGSC-trafficking process is Ca^2+^ dependent because preloading MVN neurons with the Ca^2+^ buffer BAPTA-AM, or exocytosis inhibitor TAT-NSF700, prevents the effects of BIL, indicating the upregulated activity and density of functional VGSCs as the core mechanism accountable for the BIL-induced overexcitation of neonatal neurons. Most importantly, rectification of such overexcitation with a low dose of VGSC blocker lidocaine significantly attenuates BIL-induced cell death. We suggest that this enhancement of VGSC currents directly contributes to the vulnerability of neonatal brain to hyperbilirubinemia, implicating the activity and trafficking of NaV1.1 channels as a potential target for neuroprotection in cases of severe jaundice.

## Introduction

Nearly 85% of newborns are affected with neonatal hyperbilirubinemia and clinical jaundice. Acute bilirubin (BIL) encephalopathy, if untreated, may progress to kernicterus, characterized by impairments in auditory perception, body movement, and ocular control^1,2^. Central vestibular neurons are particularly vulnerable to bilirubin-induced neurotoxicity^3–5^. MVN, the major relay station for signals to and from the cerebellum, provides the largest source and target area for the reciprocal commissural pathway to control gaze and posture^6,7^. Hyperbilirubinemia-induced neurotoxicity of MVN neurons has been linked to motor disorders observed during kernicterus, such as strabismus and gaze palsies, hypotonia, and delays in vestibular-evoked myogenic potentials (VEMP)^8–10^. Neuropathological changes such as yellow staining in vestibular nuclei are particularly notable in Gunn rat jaundice model^11^, and in the brainstem tissue from an infant with hyperbilirubinemia-induced kernicterus^12^. In developing central neurons, BIL enhances neurotransmission by increasing intracellular Ca^2+^ concentration ([Ca^2+^]_i_) and transmitter release from presynaptic terminals, as well as the excitability of postsynaptic neurons^13–15^. However, neither the identity of ion channels, nor the mechanisms underlying elevated intrinsic excitability of neonatal neurons and neurotoxicity by BIL, are known.

Voltage-gated Na^+^ channels (VGSCs) are critical for spike initiation, waveform, propagation, and firing patterns in central neurons^16^. Nav1.1 channels are widely expressed in neurons, including MVN neurons^17–19^. Studies of VGSCs have indicated that one of the major mechanisms of neuromodulation is by trafficking of channels between the cytoplasmic and cytosolic compartments^20,21^. Despite compelling evidence showing the effects of BIL on the excitability and neurotransmission, its impact on trafficking of VGSCs has never been explored.

In this study, we investigated the mechanisms underlying BIL-induced upregulation of the membrane excitability from neonatal rat MVN neurons. Not only have we found that BIL lowers the activation threshold of VGSCs but it also increases the membrane trafficking of VGSCs in Ca^2+^-dependent manner. We found that BIL-induced excitotoxicity can be attenuated by a VGSC blocker. We suggest that elevated activity and expression of VGSCs by BIL underlie increased intrinsic excitability of neonatal neurons and neurotoxicity seen in the MVN, and potentially other BIL-vulnerable brain regions.

## Results

### Bilirubin increases spontaneous firings of MVN neurons

We first performed voltage-clamp recordings from MVN neurons in brainstem slices by using cell-attached configurations to characterize physiological properties without perturbation of intracellular homeostasis (i.e., seal resistance > GΩ). In the presence of a cocktail of synaptic blockers (see the “Materials and methods” section), all neurons were spontaneously active and rhythmically fire action potentials, with each spike being registered as an inward current (*I*_inward_) followed by an outward current (*I*_outward_), which serve as the indirect readout of the inward VGSC current and the outward of potassium current, respectively.

**Fig. 1:**
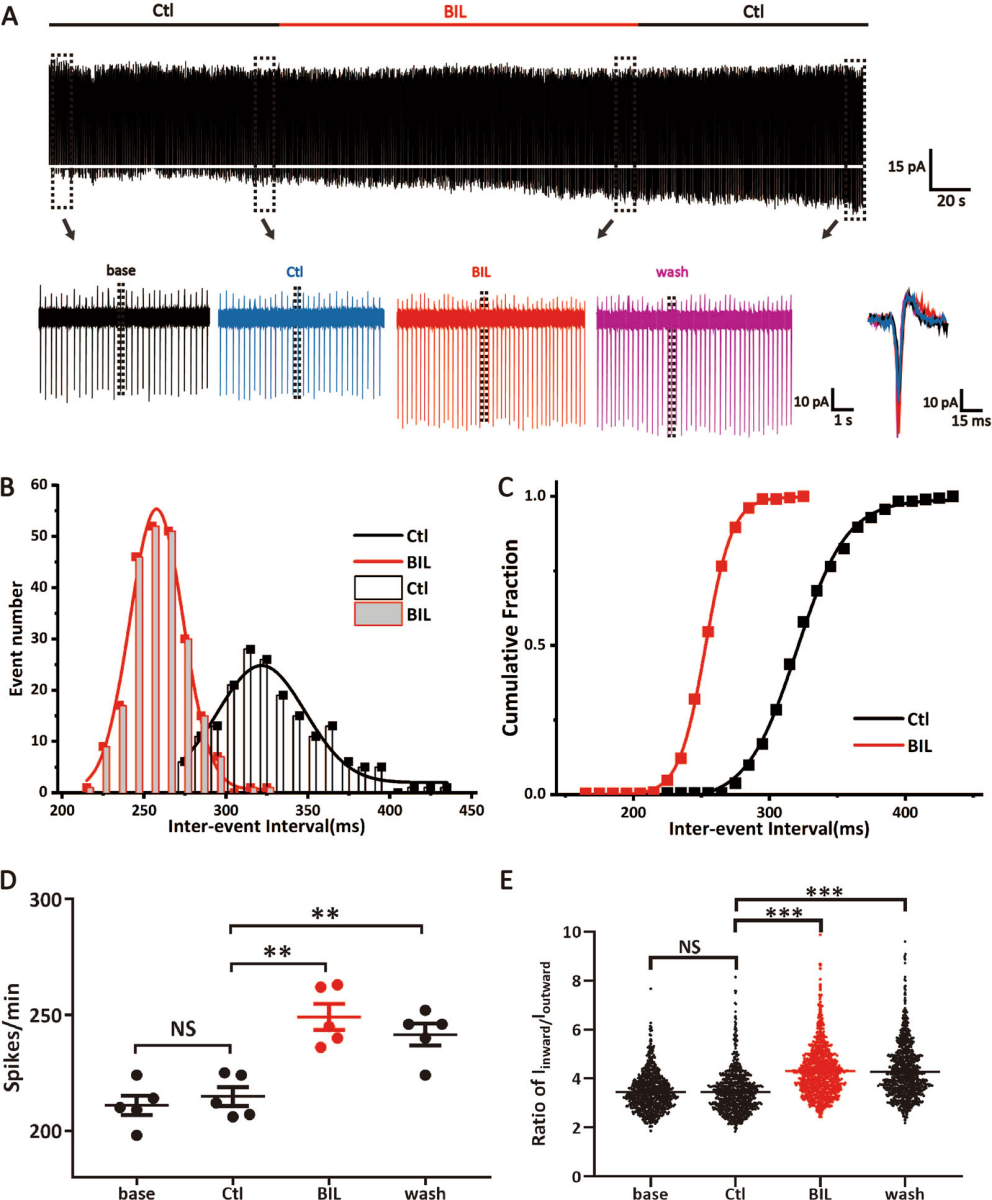
Bilirubin enhanced spontaneous firings of MVN neurons by primarily increasing the amplitude of *I*_inward_. **a** An example of cell-attached recording of spontaneous discharges from a MVN neuron, showing changes in firing frequency, *I*_inward_ and *I*_outward_ amplitude after 2 min in control buffer, 3 min in BIL buffer, and then washout. Note the enlarged amplitude of *I*_inward_ and increased firing frequency after BIL application, which appeared to be irreversible; the white dashed line refers to the main amplitude of *I*_inward_ in control buffer. Insets below show the color-coded current traces with an expanded timescale at different time points as indicated by the boxes, as well as superimposed spike waveforms during application of control (Blue) and BIL buffers (Red). **b**, **c** The event number and cumulative histograms of inter-event intervals (bin: 10 ms), showing much shorter mean intervals between events after BIL application. **d** The mean frequencies of spontaneous firings before and after BIL application, indicating a significant increase in the excitability of MVN neurons by BIL. **e** The increased ratio of *I*_inward_ and *I*_outward_ after 3 min of BIL incubation, implicating a preferential enhancement of inward current as a major cause of elevated excitability of MVN neurons. **p < 0.01, ***p < 0.001, NS not significant, one-way ANOVA with LSD post hoc test for frequency comparison and Tamhane’s T2 test for spike amplitude analysis.

To explore the acute actions of BIL on spontaneous firings, we recorded from MVN neurons in the control buffer for 2–3 min before applying BIL (3 µM), recording for another 3–5 min, and then washing out. Figure 1a shows an example, in which BIL gradually increased the firing rate, and the magnitude of *I*_inward_ and *I*_outward_, as depicted by superimposed spikes at different time points. These changes remained following washout with the control buffer. To quantify the effect of BIL on the firing rate, we measured the inter-spike intervals (ISI) of all events and plotted the data as binned and cumulative frequency histograms (Fig. 1b, c), respectively. ISI followed the normal distributions (i.e., single-component fits with the Gaussian function) and was significantly shortened by BIL as evidenced by leftward shifts in both plots (Fig. 1b, c), suggesting that these rhythmically spontaneous firings are likely driven by intrinsic pacemaker conductance(s). Both firing frequency and current amplitude were stable in the control buffer (Before: 211.00 ± 4.20 spikes/min, *I*_inward_ = 41.29 ± 0.78 pA, *I*_outward_ = 13.09 ± 0.32 pA, 1055 events from 5 neurons, After 3 min: 214.80 ± 4.09 spikes/min, *P* = 0.292, *I*_inward_ = 41.87 ± 0.76 pA, *P* = 0.447, *I*_outward_ = 13.44 ± 0.32 pA, *P* = 0.591, 1074 events from 5 neurons, Fig. 1a). Application of BIL gradually increased its firing rate (249.20 ± 5.62 spikes/min, *p* = 0.002, Fig. 1a, d), *I*_inward_ and *I*_outward_ (57.62 ± 0.94 pA, *P* < 0.001, 15.02 ± 0.32 pA, *P* < 0.001, 1246 events from 5 neurons, 5 slices). The ratio of *I*_inward_/*I*_outward_ increased from 3.46 ± 0.03 to 4.30 ± 0.03 (Fig. 1e, *P* < 0.001), indicating that this change mainly resulted from the enlarged inward current amplitude. After 3 min of washout, the excitability remained higher than control (241.60 ± 4.79 spikes/min, *P* = 0.008, *I*_outward_ = 15.57 ± 0.36 pA, *P* < 0.001, *I*_inward_ = 58.51 ± 1.00, *P* < 0.001, and *I*_inward_/*I*_outward_ = 4.27 ± 0.04, *P* < 0.001, 1208 events from 5 neurons, 5 slices). The mean ISI was significantly shortened by BIL, as revealed by quantitative analyses of either the frequency histogram (Ctl: 321.71 ± 1.98 ms, BIL: 257.63 ± 0.71 ms; 10-ms bins, from 5 neurons, 5 slices) or cumulative histogram (Ctl: 320.13 ± 0.82 ms, BIL: 253.08 ± 0.24 ms; 10-ms bins, from 5 neurons, 5 slices) (Fig. 1b, c). These results demonstrated that BIL facilitates spontaneous firings of MVN neurons by enhancing *I*_inward_, implicating VGSCs as one of the main driving forces.

BIL is light-sensitive and not stable in aqueous solution, and can be oxidized into other toxic by-products that potentially affect neuronal excitability. In order to fully ascribe the time course of bilirubin-induced changes without its possible oxidized products, we repeated the previous experiments in ascorbate acid (0.2 mM), an antioxidant. We found that ascorbate acid itself (3 min) did not have any measurable effects. However, both firing frequency and the ratio of *I*_inward_/*I*_outward_ were significantly increased after 4 min of bilirubin perfusion, and these changes were partially reversible but remained elevated even after 20 min of washing (Supplementary Fig. S1). These results suggested that even a transient surge in bilirubin itself, independent of its oxidative by-products, could induce a persistent overexcitation of MVN neurons.

### Bilirubin enhances the current mediated by VGSCs in MVN neurons

Since primary action of BIL is to enlarge *I*_inward_ in cell-attached recordings, we postulated that VGSCs were upregulated to increase in the firing rate. We made whole-cell recordings of VGSC currents (*I*_Na_) from mechanically isolated MVN neurons whose neurites were largely removed during dissociation to improve the space clamp. We lowered extracellular Na^+^ concentration (50 mM) to reduce the driving force and used a cocktail of blockers for synaptic receptors and other ion channels to ensure that the amplitude and kinetics of *I*_Na_ can be accurately measured. For this experiment, we exposed cells to 3 μM BIL buffer for >3 min, in cell-attached mode, to protect the integrity of intracellular signaling, and then broke through to establish the whole-cell voltage-clamp recording of *I*_Na_.

**Fig. 2:**
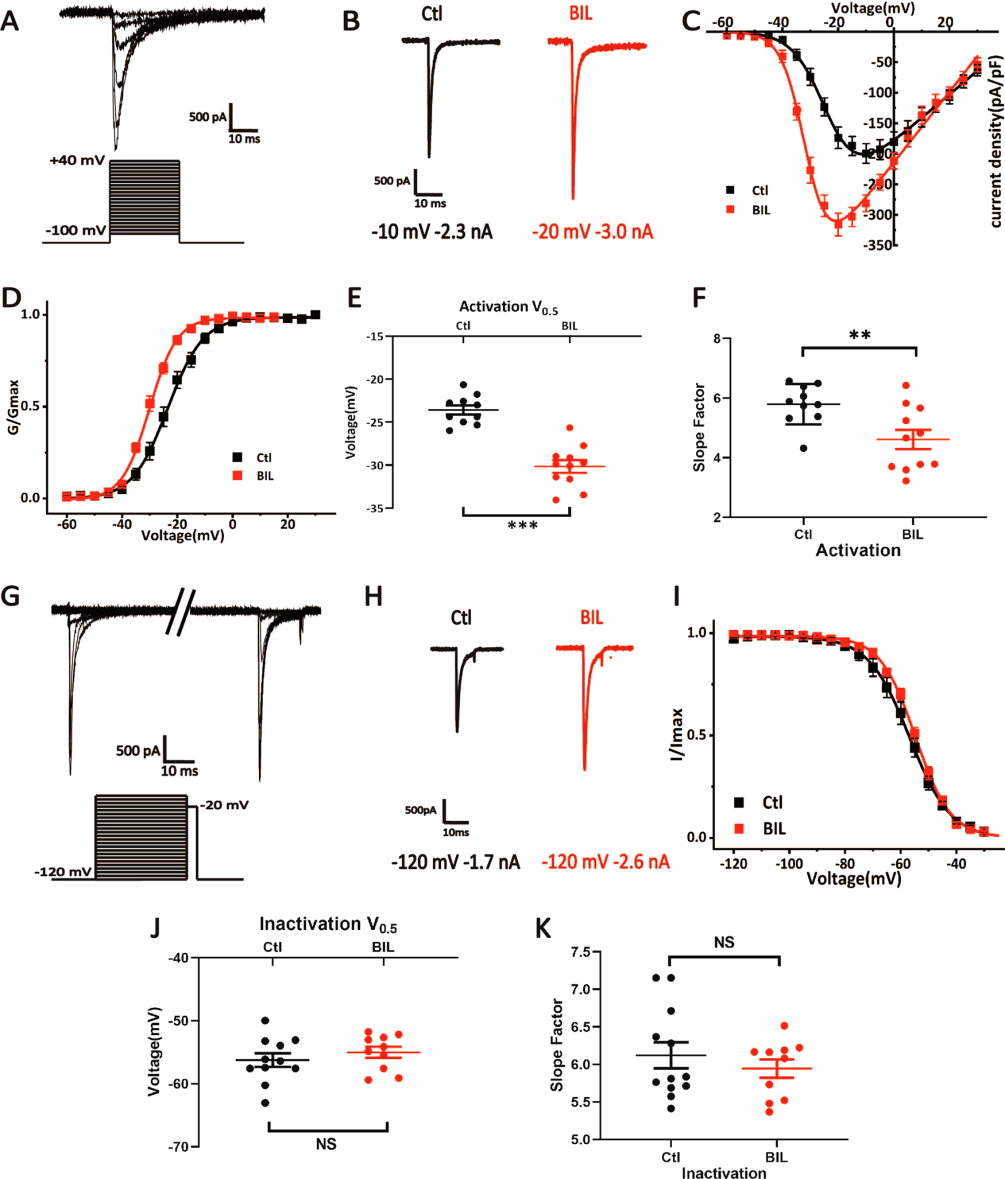
BIL enhanced VGSC currents and accelerated their activation. **a** An example of raw current trace mediated by VGSCs in response to the voltage-step protocol (below) showing a good alignment of the peak of individual currents as a result of space clamp. **b** Contrast two recording traces of maximal VGSC currents in control and BIL (3 μM), showing that BIL enlarged the amplitude and shifted the maximal activation voltage by –10 mV. **c** The current–voltage relationships (*I*–*V*) of VGSCs in control and BIL groups, showing that the current density (pA/pF) was significantly increased, while the voltage for maximal activation was hyperpolarized. **d**–**f** The overlaid activation curves of control and BIL groups are shown, as well as the pooled results of their activation *V*_0.5_ and slope factor. The BIL group showed steeper voltage dependence for activation than the control as reflected by a more negative *V*_0.5_ and a lowered slope factor. **g** Raw current traces of steady-state inactivation of VGSC currents evoked by a single test pulse to −20 mV following preconditioning voltage steps ranging from −120 to + 20 mV for 100 ms (below). **h** Example current traces showing that BIL enhanced the amplitude of VGSC current evoked by the same test pulse from −120 to −20 mV. **i** The inactivation curves of the control group and the BIL group, showing that BIL had little effects on VGSCs. **j**, **k** Pooled data showing no significant difference between control and BIL groups in inactivation *V*_0.5_ or the slope factor. **p < 0.01, ***p < 0.001, NS not significant, independent-samples *t-*test.

Figure 2a shows an example recording of *I*_Na_ activated by depolarization steps from −60 to 40 mV in 5-mV increments (holding potential: −100 mV). Figure 2b contrasts single *I*_Na_ traces from two MVN neurons with or without BIL pretreatment, showing that BIL increased the amplitude of *I*_Na_. To quantitatively compare the group results, we measured the peak amplitude of *I*_Na_ evoked by each voltage and calculated the current density for each cell (pA/pF). The pooled data were then used to construct normalized current–voltage (*I*–*V*) and conductance–voltage (*G*–*V*) curves (Fig. 2c, d). We found that the BIL group had a much larger current density than the control group (Ctl: −199.41 ± 16.04 pA/pF, 10 neurons, 10 slices vs BIL: −315.47 ± 18.64 pA/pF, *P* = 0.004, 11 neurons, 11 slices, Fig. 2c). When the *G*–*V* curves were fitted with the Boltzmann equation, we found that BIL shifted the *V*_0.5_ to a more hyperpolarized potential (*V*_0.5_: Ctl: −23.57 ± 0.54 mV vs BIL: −30.14 ± 0.73 mV, *P* < 0.001, Fig. 2e, f), and the slope factor of activation accelerated from 5.80 ± 0.21 to 4.61 ± 0.32 (*P* = 0.009, Fig. 2f). To test the influence of BIL on the steady-state inactivation of VGSCs (Fig. 2g), we delivered a series of prolonged conditioning steps (from −120 to −5 mV, 100-ms duration) before evoking *I*_Na_ with a single test pulse to −20 mV (10 ms). The amplitude of *I*_Na_ by the test pulse declined as the conditioning step being more depolarized. By plotting the normalized amplitude of *I*_Na_, we constructed steady-state inactivation curves and fit them with the Boltzmann equation (Fig. 2i). Although BIL increased the peak amplitude of *I*_Na_ (Fig. 2h), there was no significant difference between control and BIL group in all parameters for steady-state inactivation (*V*_0.5_: Ctl: −56.23 ± 1.09 mV, 11 neurons, 11 slices vs BIL: −55.00 ± 0.89 mV, *n* = 10, *P* = 0.216, 10 neurons, 10 slices, Fig. 2j; slope factor: Ctl: 6.16 ± 0.19 vs BIL: 5.94 ± 0.12, *P* = 0.433, Fig. 2k). These results indicate that BIL boosts *I*_Na_ primarily by elevating the current density of VGSCs and shifting their activation to more hyperpolarized potentials.

### BIL elevates VGSC expression in MVN neurons

**Fig. 3:**
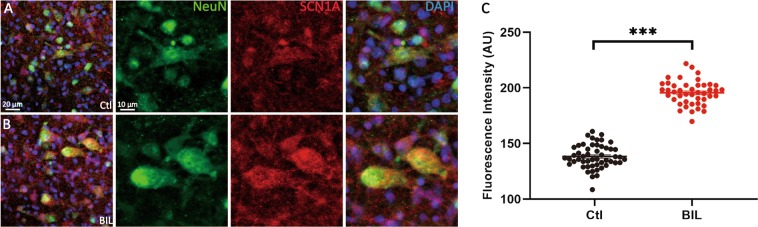
BIL elevated the expression level of SCN1A in MVN neurons. **a**, **b** Immunofluorescence labeling images of nuclei (blue), NeuN (green), and SCN1A (red) of control (**a**) and BIL group (**b**). Neurons with co-labeling of NeuN and SCN1A were chosen as AOI, and the mean fluorescence intensities of SCN1A from AOI were quantified in artificial units (AUs). **c** Statistical comparison showing larger MISCN1A in the BIL group, implicating elevated expression level of Nav1.1 after BIL treatment. ***p < 0.001, independent-samples *t-*test.

The increase in *I*_Na_ by BIL raised the possibility that BIL may upregulate the membrane expression of VGSCs in MVN neurons that are largely GABAergic^19^. We applied immunolabeling to quantify the expression level of Na_v_1.1 (encoded by *SCN1A gene*), because these channels are abundantly expressed in GABAergic neurons. Living brainstem slices (in 100 μm) containing MVN were divided into two groups with one group being treated with vehicle and the other being treated with BIL (3 µM) for 20 min before fixing the tissue with 4% paraformaldehyde. These slices were then subjected to triple labeling to identify cell nucleus (DAPI: Blue), neuron (anti-NeuN: Green), and Anti-Na_V_1.1 antibody (SCN1A: Red) (Ctl: Fig. 3a; BIL: Fig. 3b). Cells co-labeling with NeuN and SCN1A were assigned as the area of interest (AOI); the mean intensity of SCN1A (MI_**SCN1A**_) labeling of the cell membrane was calculated after correcting for background. For image acquisition during fluorescence microscopy, all parameters remained identical for both control and BIL-treated slices to minimize intertrial variability. We found that the absolute MI_SCN1A_ value of the BIL group was higher than that of the Ctl group (Fig. 3c, MI_SCN1A_: Ctl: 138.34 ± 1.47, from 52 AOIs, 7 slices, BIL: 195.55 ± 1.63, from 44 AOIs, 6 slices, *P* < 0.001), implicating that BIL could promote Na_v_1.1 expression of MVN neurons, consistent with larger *I*_Na_ in the BIL group from electrophysiological recordings.

### BIL exerts its actions in Ca^2+^-dependent manner

**Fig. 4:**
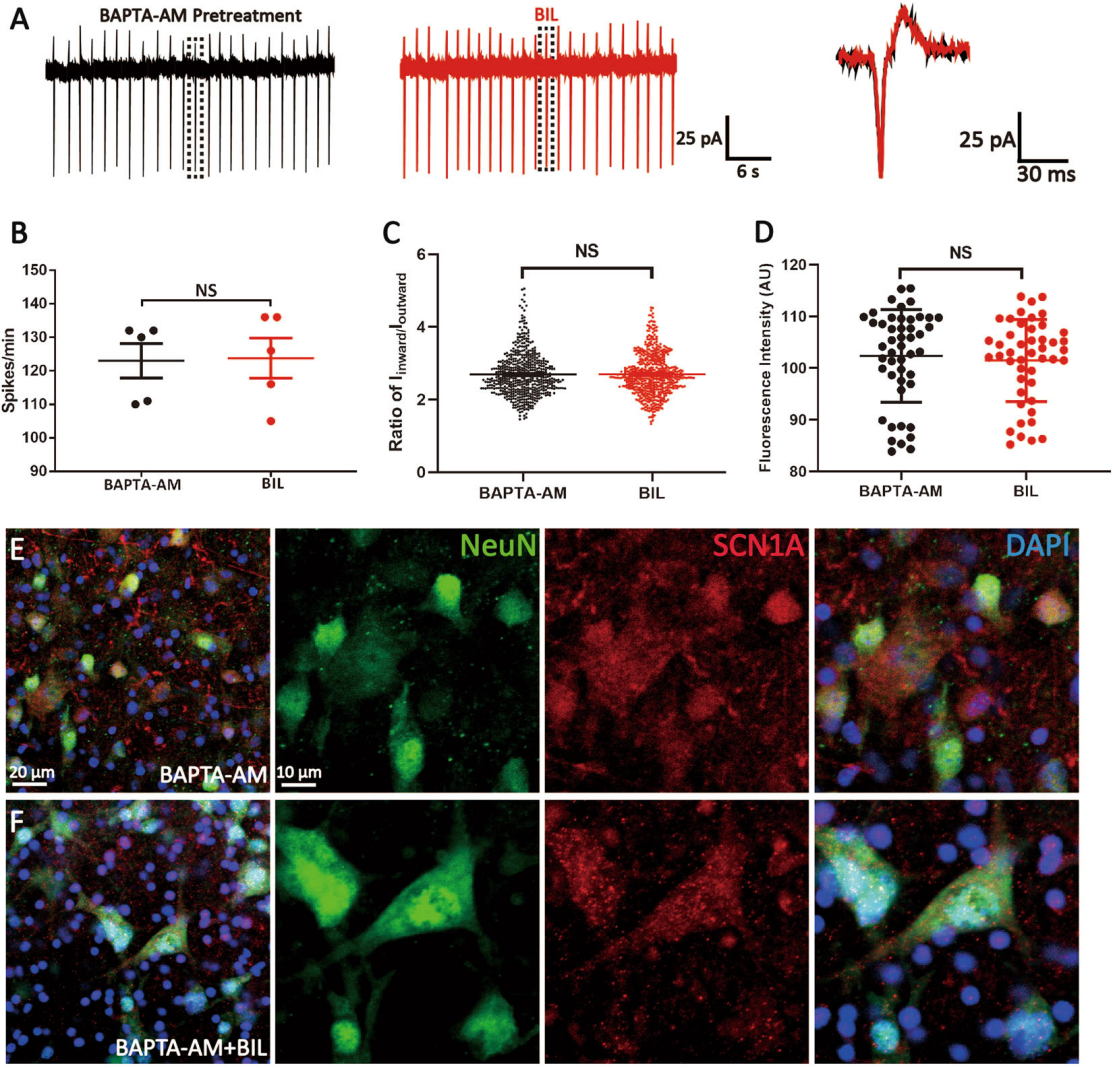
Ca^2+^ was required for BIL-induced upregulation of activity and level of VGSCs. **a** An example recording showing that neither the frequency of spontaneous firings nor the spike waveform of a MVN neuron was altered in slices preincubated in BAPTA-AM (40 μM, 30 min). **b**, **c** Statistical results showing no significant change in spike frequency, ratio of *I*_inward_/*I*_outward_. **d**–**f** MI_SCN1A_ was not statistically different between control and BIL groups (**d**) as exemplified by the immunofluorescence staining of nuclei (blue), NeuN (green), and SCN1A (red) in control (**e**) and BIL groups (**f**). NS no statistical difference, independent-samples *t* test.

BIL is known to increase intracellular Ca^2+^ concentration and augments Ca^2+^ influx through calcium channels during repetitive firings in neonatal neurons^22^. We hypothesized that elevated Ca^2+^ can act as a second messenger to trigger downstream signaling, and consequentially, upregulate the expression and elevate the activity of VGSCs. To test this, we pretreated the brainstem slices with a membrane-permeable fast calcium chelator, BAPTA-AM, and then subjected these slices for both electrophysiology and immunostaining experiments. In slices pretreated with BAPTA-AM (30 min), the firing rates were significantly downregulated, suggesting that tonic Ca^2+^ level is important for maintaining basal firings of MVN neurons (BAPTA-AM: 123.00 ± 5.12 spikes/min, *P* < 0.001). Moreover, the BIL-induced increase in the spike frequency was completely absent (BIL: 123.80 ± 5.99 spikes/min, *P* = 0.757), as were the changes in the ratio of *I*_inward_/*I*_outward_ (BAPTA-AM: 2.69 ± 0.03, 615 events from 5 neurons, 5 slices, BIL: 2.69 ± 0.02, *P* = 0.846, 619 events from 5 neurons, 5 slices, Fig. 4a–c). Immunostaining also showed that there is no difference in the MI_SCN1A_ between two groups (Fig. 4d, MI_SCN1A_: BAPTA-AM: 102.337 ± 1.31, from 47 AOIs, 6 slices; BIL: 101.478, from 46 AOIs, 7 slices; *P* = 0.657). These results indicated that Ca^2+^ is essential for BIL to increase the expression and function of VGSC.

### BIL enhances the trafficking of Na_V_1.1 to the cytoplasmic membrane

**Fig. 5:**
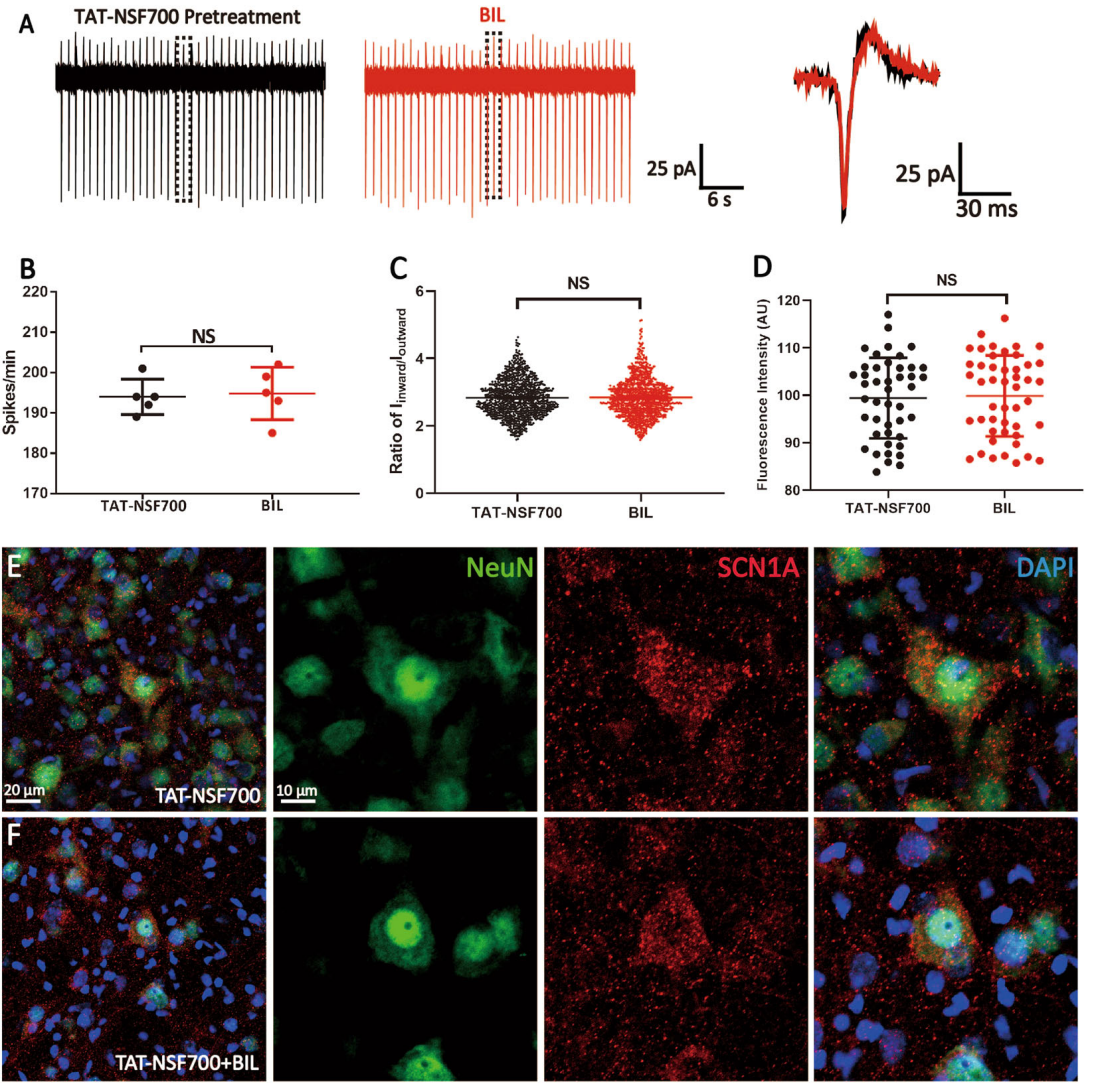
Blocking exocytosis with TAT-NSF700 precluded the effects of BIL on MVN neurons. **a**–**c** Example recording traces (**a**) showing that when the slice was preincubated with TAT-NSF700 (5 μM, 30 min), a permeable thrombin-induced exocytosis inhibitor, neither the discharge frequency of MVN neuron (**b**) nor the ratio of *I*_inward_/*I*_outward_ (**c**) was altered by BIL (3 μM, 3 min). **d–f** MI_SCN1A_ was not statistically different between control and BIL groups (**d**) in slices pretreated with TAT-NSF700, as illustrated by the immunofluorescence images comparing staining of nuclei (blue), NeuN (green), and SCN1A (red) in control (**e**) and BIL group (**f**). NS no statistical difference, independent-samples *t-*test.

The BAPTA-AM experiments raised the possibility that there is a cytosolic pool of Na_V_1.1 channels, which can be inserted onto the cytoplasmic membrane in a Ca^2+^-dependent manner to account for the BIL-induced increase in I_Na_. To address this, we pretreated MVN neurons with TAT-NSF700, a N-ethyl-maleimide-sensitive factor (NSF) inhibitor fusion polypeptide, which could prevent vesicles from transporting intracellular protein to the cytoplasmic membrane^23^. As a result of the attachment of the HIV transactivating regulatory protein (TAT), TAT-NSF700 could readily permeate the cell membrane and interact with the intracellular organelle directly. We found that 30 min of pretreatment with 5 μM TAT-NSF700 could slow the basal firing rate (TAT-NSF700: 194.00 ± 1.97 spikes/min, 5 neurons, 5 slices, *P* = 0.004), precluding BIL effects on the firing rate (BIL: 194.80 ± 2.91 spikes/min, *P* = 0.723, Fig. 5a, b) and the ratio of *I*_inward_/*I*_outward_ (Ctl: 2.83 ± 0.02 pA, 970 events from 5 neurons, 5 slices, BIL: 2.84 ± 0.02 pA, *P* = 0.633, 974 events from 5 neurons, 5 slices, Fig. 5c). These observations were validated from the immunostaining of slices preincubated with 5 μM TAT-NSF700 for 30 min and then fixed for quantitative analyses with fluorescence microscopy. MI_SCN1A_ of the TAT-NSF700 group showed no difference from that of the BIL group (MI_SCN1A_: TAT-NSF700: 99.39 ± 1.28, from 45 AOIs, 7 slices, BIL: 99.84 ± 1.25, from 47 AOIs, 6 slices, *P* = 0.816). These results suggested that BIL actively recruits Na_V_1.1 channel from the cytosolic pool to the membrane.

**Fig. 6:**
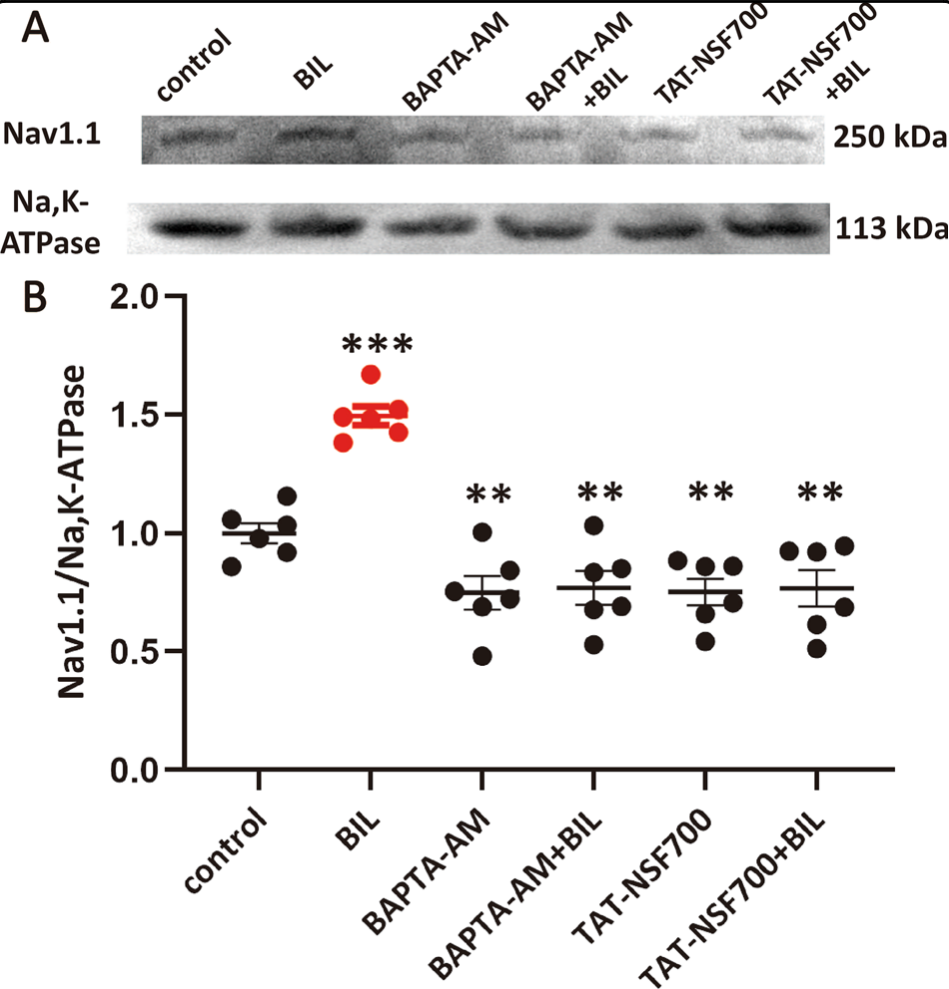
Bilirubin-induced upregulation of Na_V_1.1 channel in the cytoplasmic membrane of MVN neurons. **a** Representative immunoblots of Na_v_1.1 protein in biotinylated membrane proteins before and after BIL treatment in control, BAPTA-AM (40 μM), and TAT-NSF700 (5 μM) preincubated slices (30 min). Na, K-ATPase was used as an internal control. **b** Densitometric analyses of Na_v_1.1 were normalized to the loading control. BIL alone significantly increased the expression of Na_v_1.1, while BAPTA-AM and TAT-NSF700 could preclude its effect and reduce the basal expression of Na_v_1.1 on cellular membrane. **p < 0.05, ***p < 0.001, one-way ANOVA with LSD post hoc test.

To directly test if additional Na_V_1.1 channels were recruited to the cytoplasmic membrane by BIL, we conducted Western blotting analysis of Na_V_1.1 protein in biotinylated membrane fractions. Following pretreatments with different reagents (i.e., control, BAPTA-AM, and TAT-NSF700), slices were briefly exposed to BIL for 20 min. Membrane proteins were then biotinylated and disassociated from other cellular components before subjecting the tissue to Western blotting by using antibodies against Na_V_1.1 and Na^+^, K^+^-ATPase. We found that BIL elevated the level of Na_V_1.1 without affecting Na^+^/K^+^-ATPases on the cytoplasmic membrane, leading to a significant increase in the ratio between two proteins (Control: 1.0 ± 0.04, BIL: 1.49 ± 0.04, *P* < 0.001, *n* = 6, Fig. 6a, b). Pretreatments with BAPTA-AM and TAT-NSF700 effectively prevented such changes (BAPTA-AM: 0.75 ± 0.07, BAPTA-AM + BIL: 0.77 ± 0.07, *P* = 0.851; TAT-NSF700: 0.75 ± 0.06, TAT-NSF700 + BIL: 0.77 ± 0.08, *P* = 0.885). Notably, both BAPTA-AM and TAT-NSF700 reduced the amount of Na_V_1.1 on the cytoplasmic membrane (Ctl: 1.0 ± 0.04, BAPTA-AM: 0.75 ± 0.07, *P* = 0.026; TAT-NSF700: 0.75 ± 0.06, *P* = 0.028). These results demonstrate that both basal recycling and activity-dependent recruitment of intracellular Na_v_1.1 pool to the cytoplasmic membrane requires Ca^2+^-dependent exocytosis.

**Fig. 7:**
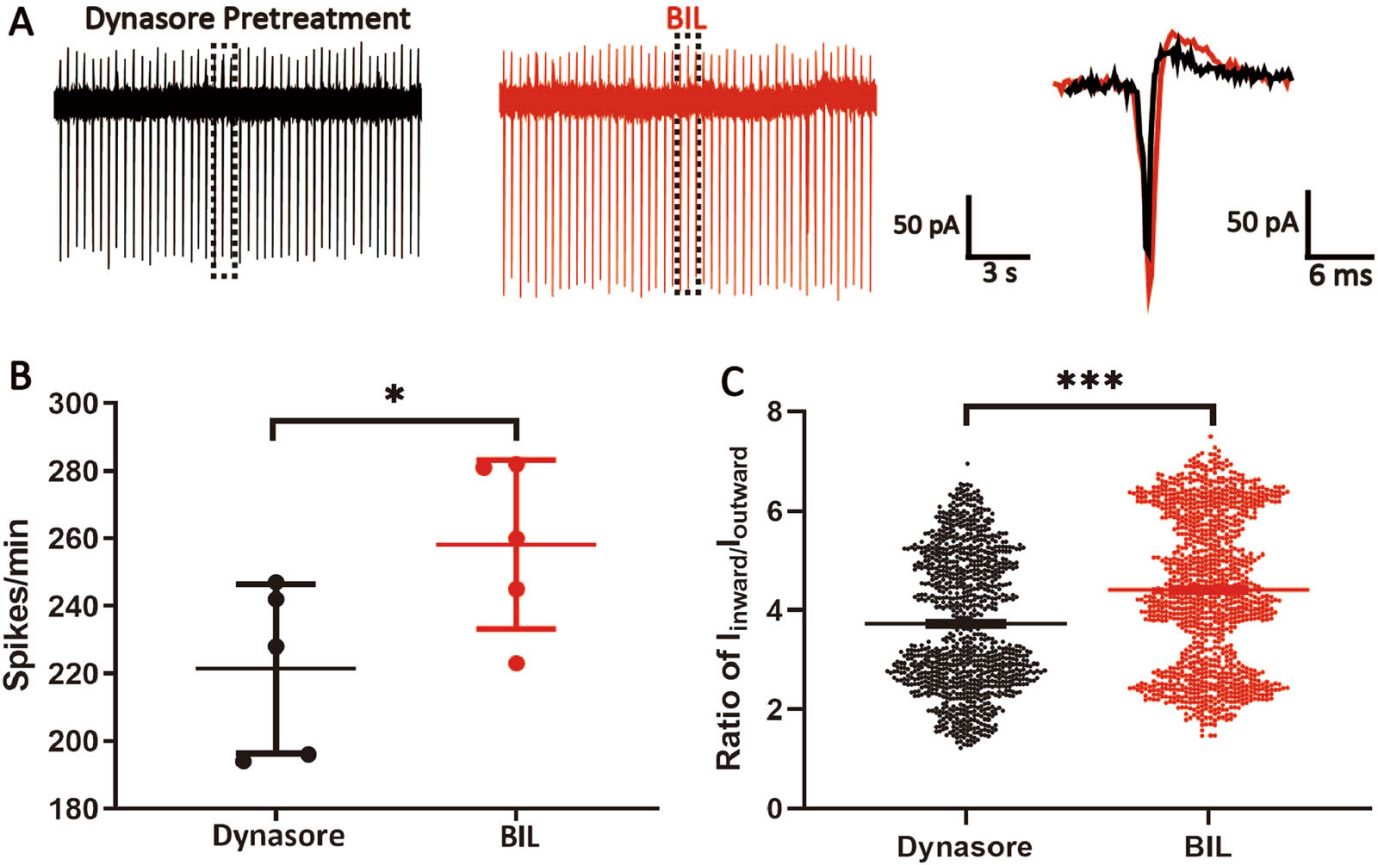
Blockade of dynamin-dependent endocytosis augments the effects of BIL on the excitability of MVN neurons. **a** An example recording showing that preincubation of slices with dynasore (40 μM, 30 min), an inhibitor for dynamin-dependent endocytosis, increased the basal firing rate of MVN neurons and further amplified BIL-induced increase in the ratio of *I*_inward_/*I*_outward_, implicating a larger sodium current. **b**, **c** Pooled data plots, summarizing the augmented increases in the firing rate and ratio of *I*_inward_/*I*_outward_. *p < 0.05, ***p < 0.001, independent-samples *t-*test.

By counteracting active membrane targeting of cytosolic Na_V_1.1 channels, a slowed endocytosis of membrane Na_V_1.1 may also potentially increase *I*_Na_. To test this, we employed dynasore, a dynamin inhibitor that can block internalization of membrane proteins. After a 30-min pretreatment with 40 μM dynasore, both basal firing rate and ratio of *I*_inward_/*I*_outward_ of MVN neurons in slices increased (Dyn: 234.40 ± 4.20 spikes/min, *n* = 5, *P* = 0.01; 3.73 ± 0.04, *P* < 0.001, 1172 events from 5 neurons, 5 slices, Fig. 7a, c). After incubation with 3 μM bilirubin, both spike frequency and ratio of *I*_inward_/*I*_outward_ also increased (264.60 ± 7.39 spikes/min, *P* = 0.011; 4.41 ± 0.05, *P* < 0.001, 1323 events from 5 neurons, 5 slices, Fig. 7b). These results demonstrated that when endocytosis is blocked, BIL can cause accumulation of VGSCs on the cell membrane in addition to its effect on the recruitment of intracellular pool to the cell membrane, thus synergistically enhancing the excitability.

### Upregulation of VGSCs induced by BIL exacerbates cell death

**Fig. 8:**
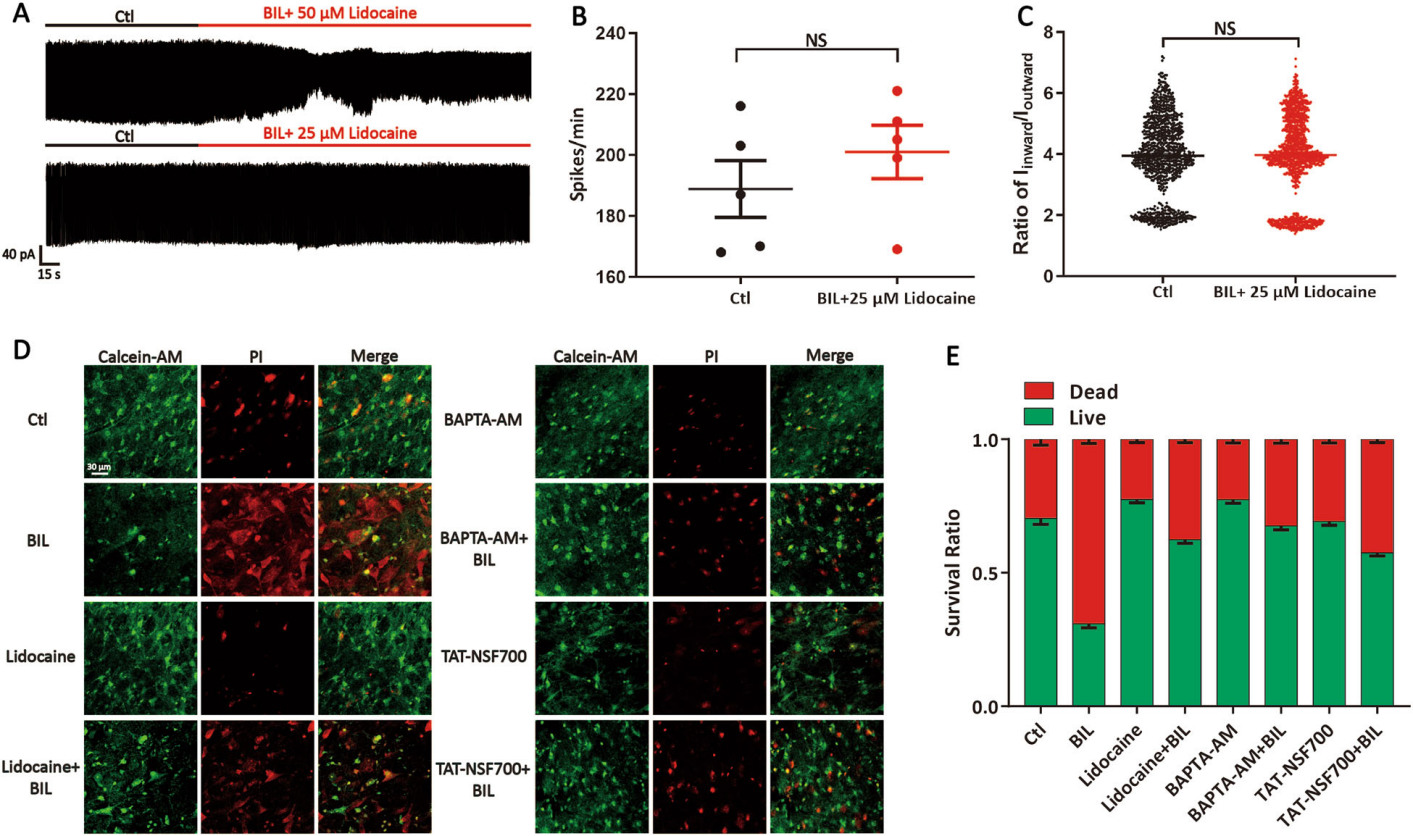
Upregulation of membrane VGSCs exacerbated MVN neuronal death. **a**–**c** Co-application of 3 μM BIL with 50 μM Lidocaine decreased both amplitude and frequency of spontaneous firings, while 25 μM Lidocaine prevented the augmenting effect of BIL on spontaneous firings without obviously changing the ratio of *I*_inward_/*I*_outward_ or frequency of spontaneous firings. **d** Representative images of Live (Calcein-AM, Green, left panel), Dead Cell (PI, Red, middle panel), and Overlay of fluorescence labeling under different experimental conditions. **e** A plot summarizing the fraction of Live (Green) and Dead cells (Red) for all experiments in (**d**). Independent-samples *t* test for spike-firing analysis, one-way ANOVA with LSD post hoc test was used for the comparison of cell death rates. NS no statistical difference.

To directly assess the influence of elevated membrane expression of VGSCs on cell vitality, we first titrated the concentrations of Lidocaine, a potent VGSC blocker, to normalize bilirubin-induced overexcitation of MVN neurons before performing cell death assays. As shown in Fig. 8a, 50 μM Lidocaine drastically decreased the amplitude and frequency of spontaneous spikes, whereas 25 μM effectively prevented the BIL-induced elevation of amplitude ratio (Ctl: 3.95 ± 0.04, Lidocaine: 3.97 ± 0.04, *P* = 0.719, from 5 neurons, 5 slices), without markedly altering the basal firing frequency (Ctl: 188.80 ± 9.30 spikes/min, Lidocaine: 201.00 ± 8.79 spikes/min, *P* = 0.226, 5 neurons, 5 slices, Fig. 8b, c). This rationalized 25 μM of Lidocaine being an adequate dose for cell vitality analysis. We conducted Calcein-AM/PI co-staining to quantify the ratio of live/dead cells, with different experimental conditions mirroring those in electrophysiological analyses (Fig. 8d). BIL resulted in significantly more cell death (Death ratio: Ctl: 30.87 ± 1.51%, BIL: 69.13 ± 1.51%, *P* < 0.001, 10 images from 5 slices), while Lidocaine, BAPTA-AM, and TAT-NSF700 could effectively attenuate BIL-induced cell death (Death ratio: BIL: 69.13 ± 1.51%, Lidocaine + BIL: 37.60 ± 1.32%, *P* < 0.001, BAPTA-AM + BIL: 32.50 ± 1.49%, *P* < 0.001, and TAT-NSF700 + BIL: 42.51 ± 1.20%, *P* < 0.001, 10 images from 5 slices, Fig. 8d, e). Interestingly, comparing the control groups, Lidocaine and BAPTA-AM exhibited less basal death (Death ratio: Ctl: 30.87 ± 1.51%, Lidocaine: 22.57 ± 1.25%, *P* = 0.012, BAPTA-AM: 22.59 ± 1.40%, *P* = 0.018, 10 images from 5 slices), indicating that suppression of excitability and buffering Ca^2+^ could inhibit neurotoxicity.

## Discussion

**Fig. 9:**
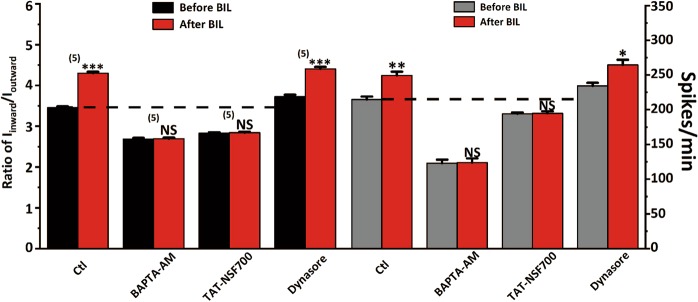
Plots summarizing the effects of BIL on the ratio of *I*_inward_/*I*_outward_ (left) and the firing rate (right) under different pretreatment conditions. Each pair of bars (black/gray vs red) represents the results before and after BIL application in the same population of neurons (*n* values are given in brackets above) with pairwise student *t* tests indicated by asterisk symbols being statistically significant. NS denotes no statistical difference. Dashed lines in each plot represent the basal levels without any manipulations.

**Fig. 10:**
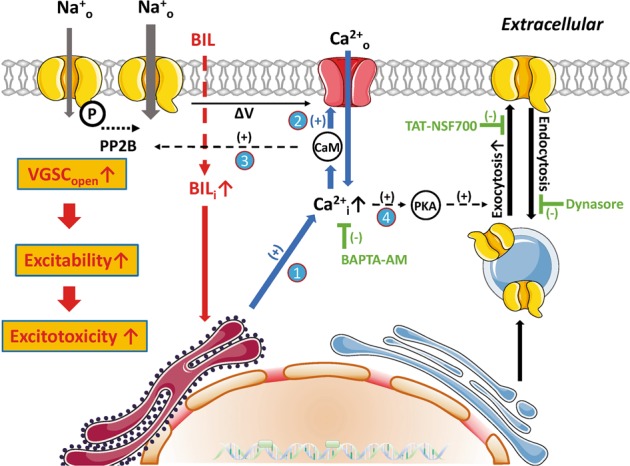
Work model summarizing Ca^2+^-dependent signaling pathways and feed-forward loop activated by BIL to elevate the neural excitability. (1) BIL penetrates the cytoplasmic membrane and induces Ca^2+^ release from internal stores; Ca^2+^ serves as a second messenger to (2) enhance Ca^2+^/CaM-dependent facilitation of VGSCs and augment Ca^2+^ influx; (3) activate Ca^2+^/CaM-dependent protein phosphatase to dephosphorylate VGSCs and enhance their gating, then further promote Ca^2+^ influx; (4) activate protein kinase A to facilitate phosphorylation-dependent membrane targeting of cytosolic pool of VGSCs. These pathways form a feed-forward loop to boost the excitability of neonatal MVN neurons.

The principal finding is that BIL upregulates the firing frequency of neonatal MVN neurons by enhancing *I*_Na_ via Ca^2+^-dependent recruitment of Na_V_1.1 channels to the cytoplasmic membrane (Fig. 9). There appears a positive feed-forward loop, through which BIL first elevates intracellular Ca^2+^ and activates multiple Ca^2+^-dependent pathways to boost neuronal excitability and spontaneous firings, which further amplify intracellular Ca^2+^ level and trafficking of VGSCs (Fig. 10). BIL leads to overexcitation and Ca^2+^ overload, beyond the capacity of endogenous buffers to cope with, ultimately resulting in excitotoxicity. In summary, brief exposure of bilirubin to MVN neurons exerts fast and persistent effects on excitability by upregulating the density and activity of Na_V_1.1 channels (Supplementary Fig. S1), while prolonged exposure to BIL aggravates cell death. Our results provide evidences for Na_V_1.1 being a key player linking the acute and chronical effects of bilirubin, implicating these channels as the potential target for intervention, or prevention, of hyperbilirubinemia-induced kernicterus.

The pathologically relevant concentrations of free bilirubin remain elusive, in part due to the mismatch in timing of biochemical measurements of bilirubin and the onset of symptoms in human neonates. However, previous studies^24,25^ from a widely accepted model, Gunn rat acute bilirubin encephalopathy model, showed that free bilirubin was 2743–10080 (in nM) of sulfadimethoxine-treated j/j pups. These numbers are in line with our choice of bilirubin concentrations for its acute actions. Our study differs in its objective and experimental paradigms from others that focus on the chronic effects of bilirubin by using lower doses for extended periods^26,27^.

BIL can enhance presynaptic neurotransmitter release, induce Ca^2+^ release from internal stores, and Ca^2+^/CaM-dependent facilitation of Ca^2+^ channels in postsynaptic neurons^2,14^. Since our experiments were performed in the presence of synaptic receptor antagonists, mechanisms underlying the enhancement of spontaneous firings and *I*_inward_ induced by BIL must work through Ca^2+^ signaling in postsynaptic loci, as is supported by experiments with Ca^2+^ chelator BAPTA-AM. The attenuated basal firing rate and *I*_inward_/*I*_outward_ ratio by BAPTA-AM and TAT-NSF700 suggest that a relatively high intracellular Ca^2+^ level is required to support spontaneous firings by dynamically regulating recycling and trafficking of Na_V_1.1 protein. Although BIL-induced upregulation of membrane expression and function of VGSCs contributes to the increase in the firing rate, VGSCs are unlikely the sole determinant of spontaneous discharges of neonatal MVN neurons. These neurons fire rhythmically with ISI following the normal Gaussian distribution, indicating that there are active pacemaker conductances (e.g., HCN1 channels^28^) driving spike discharge. Therefore, the effects of BAPTA-AM and TAT-NSF700 on basal firings could be attributed to multiple ion channels, among which VGSCs are the key contributor to the global excitability of neonatal MVN neurons.

The effect of BIL on VGSCs appears to be multifaceted. BIL significantly shifted the voltage dependence of activation toward more hyperpolarized potentials and increased the current density. BIL can activate Ca^2+^-dependent post-translational modifications of VGSCs on the membrane and/or insertion of a pre-existing pool of VGSCs. A lack of phospho-specific antibody against Na_V_1.1 channels precluded us from directly assessing their phosphorylation dependence, but future investigations shall address exactly how such modifications underlie both BIL-induced gating and trafficking of Na_V_1.1 channels. Nevertheless, activation of Ca^2+^/CaM-dependent phosphatase 2B can dephosphorylate VGSCs and enhance their current amplitude, while activation of cAMP-dependent protein kinase can phosphorylate VGSCs to promote their trafficking to the membrane^29,30^. BIL can also lead to widespread inhibition on serine/threonine protein kinase-dependent protein/peptide phosphorylation, albeit at much higher concentrations^31^, raising the possibility that direct inhibition of protein kinases might regulate these channels. We rationalized our results that the immediate action of BIL is to activate intracellular signaling pathways to rapidly promote the gating of VGSCs but rather slow down their membrane targeting. The fact that BIL-induced increase in *I*_inward_ develops slowly, and this can be further amplified when dynamin-dependent internalization was blocked, lending strong support to such an interpretation. Finally, we demonstrated that the upregulation of cytoplasmic membrane VGSCs directly contributes to overexcitation of MVN neurons and exacerbates neurotoxicity, which can be effectively rectified by Lidocaine, BAPTA-AM, or TAT-NSF700. Thus, we conclude that BIL works through multiple pathways to synergistically boost the excitability and firings of MVN neurons in a positive feed-forward cycle that exacerbates cell death.

Taken together, we propose a working model to integrate our findings as depicted (Fig. 10). BIL, a membrane-permeable substance, can induce Ca^2+^ release from internal stores such as the endoplasmic reticulum and mitochondrial membranes, and augment Ca^2+^/CaM-dependent facilitation of VGSCs^2^, while spontaneous firings further boost intracellular Ca^2+^ level to activate protein kinases and phosphatases, which, in turn, upregulates the membrane localization and gating of VGSCs. These effects converge to excessive firings and Ca^2+^ overload, eventually leading to the excitotoxicity.

MVN neurons in brainstem are particularly vulnerable to BIL-induced neural injury during hyperbilirubinemia, and in severe cases, permanent vestibulo-motor deficits^4,10^. It is believed that the stability of firing patterns in MVN neurons is critical for sensory signal conduction and integration, and demands a dynamically fine-tuned balance of ion channels to maintain the proper firing frequency^32^. Our results demonstrate that BIL activates the proposed positive feed-forward loop to disrupt the balance by primarily targeting VGSCs in neonatal MVN neurons. Bilirubin can also elevate the excitability of neurons in the cochlear nucleus^15^, another brainstem region that is known to be vulnerable to hyperbilirubinemia-induced toxicity. Our conclusion is generalizable and warrants future studies to evaluate the impact of BIL on other subpopulations of brainstem neurons. It can be envisaged that chronic elevation of bilirubin, if not timely treated, may cause excessive excitation and Ca^2+^ overload to kill neonatal neurons. Thus, our study implicates VGSCs and their trafficking as potential molecular and cellular targets for protection and intervention against hyperbilirubinemia-induced neurotoxicity.

## Materials and methods

### Ethical approval

Experiments were conducted in conformity with the institutional principles for the care and use of animals, and experimental protocols were approved by the Ethics Committee of the Sixth People’s Hospital of Shanghai and Shanghai Jiao Tong University. Throughout the experiment, all efforts were carried out to minimize animal suffering.

### Preparation of MVN slices and isolated neurons

Brainstem slices containing MVN were obtained as previously described^33^. In brief, postnatal day 4–6 Sprague Dawley rats were euthanized by decapitation under anesthesia with sodium pentobarbital (55 mg/kg, i.p.). Brains were then quickly immersed in oxygenated and ice-cold artificial cerebrospinal fluid (aCSF) containing (in mM) 124 NaCl, 5 KCl, 1.2 KH_2_PO_4_, 2.4 CaCl_2_, 1.3 MgSO_4_, 24 NaHCO_3_, 5 HEPES, and 10 glucose, with the pH being adjusted to 7.3 (osmolality of 300–310 mOsm). Next, the brainstem containing the MVN was sectioned into transverse slices (300-μm thick) by using a vibratome (VT-1200S, Leica, Germany). Brainstem slices were subsequently incubated in aCSF saturated with 95% O_2_ and 5% CO_2_ for 30 min at 35–37 °C, and slices were maintained at the room temperature for recordings. Single cells were mechanically obtained from brainstem slices by using a homemade device equipped with fire-polished glass pipettes, oscillating at 50 Hz in the close proximity of the MVN^34^. MVN neurons were dissociated by fluid turbulence and dispersed onto 35-mm-diameter dishes containing standard solution comprising (in mM) 150 NaCl, 5 KCl, 2 CaCl_2_, 1 MgCl_2_, 10 glucose, and 10 HEPES. The pH of the solution was adjusted to pH 7.4 with Tris base.

### Reagents

Reagents including free bilirubin, dynasore, cycloheximide, BAPTA-AM, Lidocaine, 6,7-dinitroquinoxaline-2,3-dione (DNQX), D-2-Amino-5-phosphonopentanoic acid (D-AP-5), bicuculline, and strychnine were purchased from Sigma (St. Louis, MO), and TAT-NSF700 was obtained from Anaspec, Inc. Bilirubin was first dissolved in 0.1 M NaOH to a concentration of 1 mM, stored in single-use dose in the dark at −20 °C (for <24 h), and diluted to a final concentration of 3 μM immediately before use. All the aCSF reservoirs were shielded with tinfoil to minimize light-induced oxidation of bilirubin, and no colloid or precipitation was observed during all the processes. The final concentrations of dynasore, cycloheximide, TAT-NSF700, BAPTA-AM, and Lidocaine were 40 μM, 25 μM, 5 μM, 40 μM, and 25 μM, respectively. Neurons were exposed to different reagents through a square-tube gravity perfusion system.

### Electrophysiology of brainstem slices and dissociated cells recordings of spontaneous firings in MVN slices

The MVN was visually identified in brainstem slices with a 60× water immersion objective attached to an upright microscope (ECLOPSE TE-2000U, Nikon, Japan). Spontaneous action potential currents were recorded from MVN neurons with cell-attached patch-clamp technique by using an amplifier (EPC-10 usb; HEKA, Germany), under voltage-clamp mode with pipette potential of −70 mV. Patch electrodes were pulled from borosilicate capillary glass by using a vertical pipette puller (PC-10; Narishige, Japan) and had a resistance of 4–7 MΩ. The pipettes were filled with an intracellular solution containing (in mM) 97.5 K-gluconate, 32.5 KCl, 0.5 ethylene glycol tetraacetic acid (EGTA), 40 4-(2-hydroxyethyl)-1-piperazineethanesulfonic acid (HEPES), and 1 MgCl_2_ (adjusted to pH 7.2 with Tris base), while the extracellular solution contained aCSF with DNQX (40 μM), D-AP-5 (50 μM), bicuculline (10 μM), and strychnine (1 μM) to block glutamatergic and glycinergic synaptic currents. Data were recorded and filtered at 1–3 kHz and sampled at 3–10 kHz by using a Dell computer equipped with PatchMaster software (HEKA).

### Voltage-clamp recordings of sodium currents

Dissociated neurons were bathed in external standard solution and visualized by using the phase-contrast mode of an inverted microscope (TE-2000U; Nikon, Japan). Whole-cell voltage-clamp recordings were made on a single cell with an amplifier (EPC-10; HEKA, Germany). Series resistance varied from 5 to 10 MΩ; all reported results were obtained from recordings in which ~90% of the series resistance could be compensated. Cells showing higher resistances were omitted from the analysis. Patch electrodes were acquired as previously described and had a resistance of 4–7 MΩ. The patch pipette solution used to record VGSCs contained (in mM) 140 CsCl, 10 TEA-Cl, 10 EGTA, 10 HEPES, and 1 MgCl_2_·6H_2_O (adjusted to pH 7.2 with Tris base), and the extracellular solution to block K^+^ and Ca^2+^ currents contained (in mM) 50 NaCl, 5 KCl, 2 CaCl_2_, 1 MgCl_2_, 10 HEPES, 10 Glucose, 90 TEA-Cl, and 10 4-AP, 0.1 with CdCl_2_. The lowered concentration of sodium was used to reduce the voltage-clamp error owing to the large amplitude and fast kinetics of Na^+^ current observed in MVN neurons, whereas TEA-Cl and 4-AP were used to inhibit potassium currents in MVN neurons. Current density was determined by dividing current by cell membrane capacitance (pA/pF). All signals were filtered at 1–3 kHz, sampled at 3–10 kHz by using a Dell computer equipped with PatchMaster software (HEKA), and collected with the aid of the patch-clamp amplifier. All experiments were performed at room temperature (23–27 °C).

Currents mediated by VGSCs were recorded in voltage-clamp mode at a holding potential of −100 mV and were activated by step depolarization from −60 to 40 mV in 5-mV increments, by using test pulses 50 ms in duration (Fig. 2a). Current–voltage (*I*–*V*) curve was fit with equation: *I* = (*V*–*V*_rev_)×Gmax/(1 + $e^{(V_{m}-V_{0.5})/\mathrm{dx}}$) and *V*_rev_ (the reversal potential for sodium channels) was calculated and used in the equation: ***G*** = *I*/(*V*_m_–*V*_rev_) to acquire the conductance value of each test voltage; then activation curves were obtained by fitting the data points with a Boltzmann equation in the form: *G*/*G*max = (*A*_1_–*A*_2_)/(1 + $e^{(V_{m}-V_{0.5})/\mathrm{dx}}$) + *A*_2_, where *G*_MAX_ is the maximal peak conductance, *G* is the peak conductance at each test voltage, *V*_0.5_ is the potential of half-maximal activation, dx is the reciprocal of slope factor, and *V*_m_ is the membrane potential. *A*_1_ and *A*_2_ represented the initial and final value of conductance. In the episodic stimulus protocol, used to evaluate the voltage dependence of steady-state inactivation, preconditioning steps, ranging from −120 to −5 mV for 100 ms, preceded the single test pulse at −20 mV for 10 ms (Fig. 2g). Steady-state inactivation curves were obtained by fitting the data points with a Boltzmann relationship in the form: *I*/*I*_MAX_ = (*A*_1_–*A*_2_)/(1 + $e^{(V_{m}-V_{0.5})/\mathrm{dx}}$) + *A*_2_, where *I*/*I*_MAX_ is the relative current, *V*_0.5_ the voltage at which half-maximal inactivation is reached, and dx the reciprocal of slope factor. The reversal potential for sodium current was measured experimentally from each neuron.

### Immunofluorescent staining and image acquisition

Brainstem slices were sectioned at a thickness of 100 μm with a vibratome and then were incubated separately in 15-ml tubes filling with aCSF (Ctl group) or aCSF + 3 μM BIL (BIL group) for 20 min; both tubes were continuously bubbled with 95% O_2_ and 5% CO_2_. In experiments described in Figs. 3–5, preincubation with specified reagents (BAPTA-AM, etc.) preceded the exposure to BIL. Then slices were washed with aCSF 3 times for at least 3 min, and then immersed in 4% paraformaldehyde for 30 min at room temperature. Following 3 rinses in cold PBS, permeabilization in 0.3% Triton X-100, and blocking with 10% goat serum and 1% BSA (bovine serum albumin) for 1 h, the slices were then incubated overnight at 4 °C in primary antibody (1:50, rabbit anti-SCN1A IgG, 20 μg/ml, Abcam; 1:50, mouse anti-NeuN IgG, 20 μg/ml, Abcam). After 3 rinses in cold PBS, slices were incubated for 1 h with the appropriate secondary antibodies conjugated to Alexa 488 (1:500, goat anti-mouse IgG, 2 μg/ml, Abcam) and Alexa 594 (1:500, goat anti-rabbit IgG, 2 μg/ml, Abcam). Slices were mounted with a coverslip by using Fluoroshield with DAPI (Abcam).

To quantify the expression of Na_V_1.1 in MVN neurons, we acquired Z-stack confocal images (0.3 μm apart over 5–10 μm) with a Zeiss LSM-710 microscope (Carl Zeiss Microimaging, Germany) by using a ×40 objective lens. DAPI (EX: 408 nm), Alexa 488 (EX: 488 nm), or Alexa 594 (EX: 594 nm) was visualized via blue-violet diode, Argon-ion, and Green HeNe excitation, respectively. The periphery of cells co-labeled with NeuN and SCN1A was chosen as AOI (area of interest), carefully marked with polygon tool by Zen 2011 software (Carl Zeiss), and then the mean fluorescence intensity of SCN1A immunolabeling in the AOI could be directly calculated by Zen 2011 software. The same process was conducted on randomly chosen areas between cells, and the mean fluorescence intensity was subtracted as background.

### Membrane surface protein biotinylation and immunoblotting

Brainstem slices were sectioned at a thickness of 500 μm with the vibratome and then divided into three paired groups (Control vs BIL) preincubated for 30 min with control aCSF, BAPTA-AM, or TAT-NSF700, respectively, before a 20-min exposure to BIL. Slices were washed with aCSF 3 times for at least 3 min and subjected to biotinylation treatment as previously described^35^. Briefly, slices were gently transferred to ice-cold bubbled aCSF containing 1 mg/mL sulfo-NHS-SS-biotin (Pierce) for 45 min followed by immersion into a quenching solution for 5 min. Slices were then rapidly rinsed with phosphate-buffered saline (PBS, in mM: KH_2_PO_4_ 1.06, NaCl 155.17, and Na_2_HPO_4_ 2.97, pH 7.4) followed by Tris-buffered saline (TBS, in mM: Tris-HCl 50, NaCl 150, pH 7.6), both supplemented with protease cocktail inhibitor (1:250, Halt cocktail, Pierce), and homogenized in a lysis buffer (Pierce) supplemented with 1× protease inhibitors, solubilized at 4 °C by using rotation and intermittent vortexing for 1 h, and centrifuged at 10,000 × *g* for 2 min. The supernatants were incubated with a neutravidin agarose bead slurry column (Pierce) and rotated for 3 h at 4 °C to bind surface-biotinylated proteins, and then the beads were washed 3–4 times in the presence of protease inhibitors (1:250). The products of the first wash were saved as the flow-through fractions, which were collected by centrifugation at 1000 × *g* for 3 min and presumed to predominantly contain unbound cytoplasmic and internal proteins. The beads were finally incubated with SDS–PAGE sample buffer supplemented with 53 mM DTT for 1 h at 37 °C to elute biotinylated proteins. Samples were heated at 95 °C for 3–5 min, centrifuged at 4 °C for 2 min, and migrated on sodium dodecyl sulfate–polyacrylamide gels (SDS–PAGE).

Samples containing target protein that were resolved on SDS–PAGE were transferred onto polyvinylidene difluoride membranes (Millipore, Billerica, MA, USA) as previously described^36^. The membranes were blocked with 5% nonfat milk in Tris-buffered saline supplemented with Tween (TBST) for 2 h at room temperature. They were then incubated overnight at 4 °C with primary antibodies: rabbit anti-SCN1A (1:500, BosterBio Inc., CA, USA), rabbit anti-Na, K-ATPase (1:5000, Abcam Inc., MA, USA). All primary antibodies were diluted in 5% nonfat milk in TBST. The membranes were then washed with TBST and incubated for 2 h at room temperature with the corresponding horseradish peroxidase-conjugated secondary antibodies. Enhanced chemiluminescence reagent (Thermo Fisher Scientific, Rockford, IL, USA) was used to detect the signal, and ChemiDoc XRS + (Bio-Rad, Hercules, CA, USA) was used to visualize the image. The results were analyzed and quantified by Image-J software (version 1.52i, NIH, Bethesda, MA, USA); Na, K-ATPase was chosen as loading control. Western blot analyses were performed six times to acquire pooled results in Fig. 6.

### Assessment of cell vitality with Calcein-AM/PI co-staining

Brain slices containing MVN were dissected at 50 μM with a vibratome, pretreated as previously described, then incubated with 1 μM Calcein-AM and 2 μM PI (Propidium Iodide, Solarbio, China) for 1 h at 37 °C, and washed with aCSF 3 times. Slices were observed with confocal microscopy (LSM-710, Zeiss, Germany) after fixing for 40 min by 4% paraformaldehyde (Solarbio, China). The live and dead cells were determined by microscopic examination at 40× magnification. The number of staining was measured with the help of Image-J software (version 1.52i, NIH, Bethesda, MA, USA).

### Data analysis

All data are stored on a personal computer for further off-line analysis. VGSCs were measured by either current size (pA) or current density (pA/pF); PatchMaster v2x73.5 (HEKA Elektronik), Clampfit 10.2 software (Molecular Devices), Origin 9.3 (Microcal Software), MiniAnalysis Program (Synaptosoft, NJ, USA), and Adobe Illustrator CC (Adobe System Inc.) were used for data analysis and graphic representation. Statistical analyses were performed by using SPSS 22.0 software (SPSS Inc.). Values in the text and figures were expressed as means ± SEMs. Independent-samples *t-*test or paired Student’s *t*-test were used for pairwise comparisons. When multiple groups were compared, one-way analysis of variance (ANOVA) was used with the least significant difference (LSD) or Tamhane’s T2 post hoc test according to the Levene’s test to determine intergroup differences in the presence of an overall significant difference. A *p* < 0.05 was interpreted as being significant.

## Supplementary information

Supplementary material

Supplementary material
