# Supplementary material for: Ca2+-dependent recruitment of voltage-gated sodium channels underlies bilirubin-induced overexcitation and neurotoxicity
Source: Cell Death Dis. 2019 Oct 10;10(10):774. doi: 10.1038/s41419-019-1979-1 (PMC6787254; doi:10.1038/s41419-019-1979-1)
Supplement: Supplementary file 2 — Supplementary material [file 41419_2019_1979_MOESM2_ESM.docx]

In order to fully ascribe the time course of bilirubin-induced changes while excluding the effect of oxidized products from bilirubin, we repeated experiments as in Figure 1 in the presence of ascorbate acid (0.2 mM) while washout period being extended to 20 min. 3 min perfusion with ascorbate acid alone wouldn’t change neither the firing frequency nor the ratio of I_inward_/I_outward_ (**Frequency:** base:246.4± 7.49 spikes/min, control: 247.2±5.50 spikes/min, P=0.868, **Ratio**: base: 4.83±0.05, control: 4.84± 0.08, P=0.848, 5 neurons from 5 slices). However, both firing frequency and ratio of I_inward_/I_outward_ were significantly increased after 4 min bilirubin perfusion (**Frequency:** control: 247.2±5.50 spikes/min, BIL: 314.20± 4.77 spikes/min, P<0.001, **Ratio**: control: 4.84±0.08, BIL: 5.76±0.05 P<0.001, 5 neurons from 5 slices), and these changes are partially reversible but remain elevated even after 20 min washing (**Frequency:** control: 247.2±7.45 spikes/min, Washing 20 min: 269±3.81, P<0.01, **Ratio**: control: 4.84±0.08, Washing 20 min: 5.18±0.05, P<0.001, **Supplementary Figure S1**). These results implicate that even a transient surge in bilirubin itself, independent of its oxidative by-products, could induce a relative persistent overexcitation of MVN neurons and neurotoxicity.


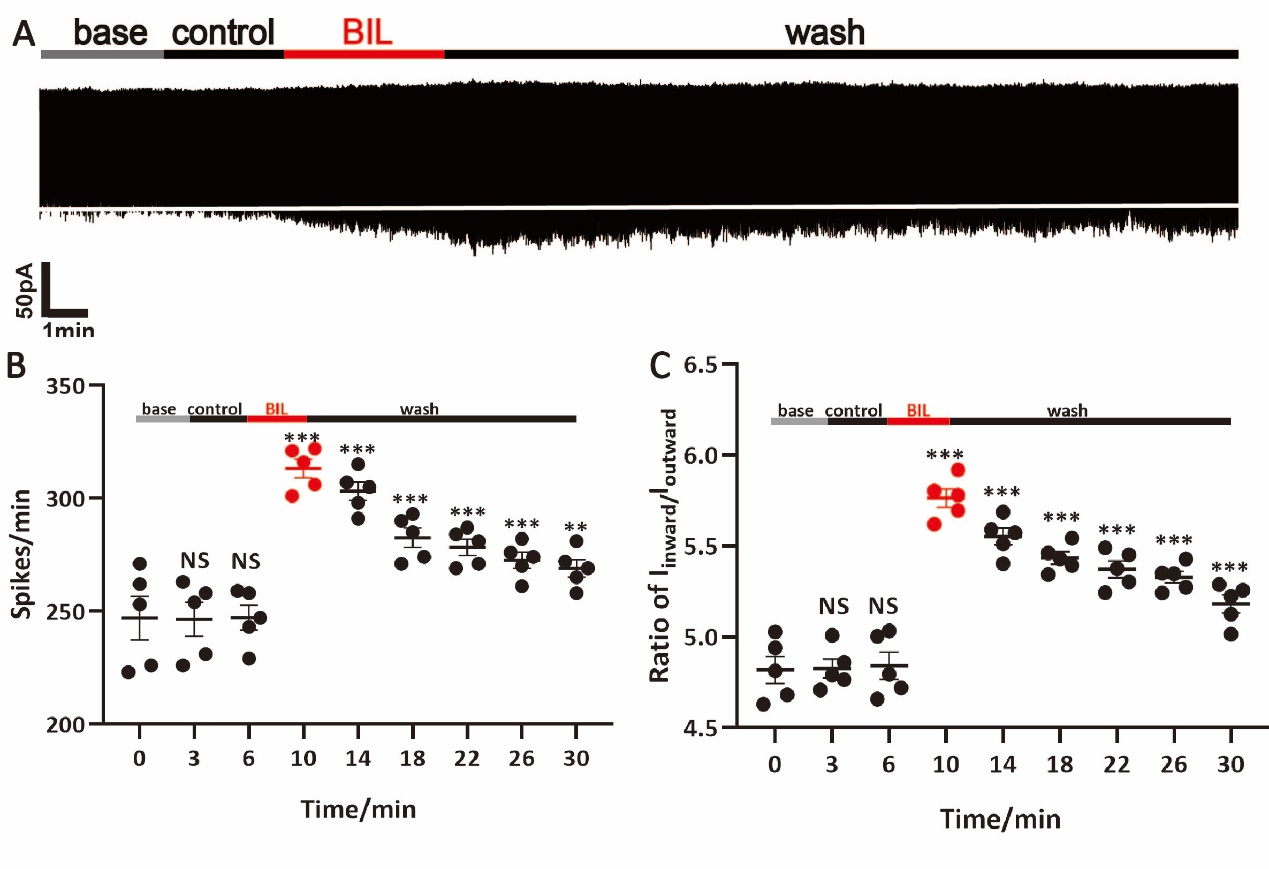


**Figure S1.** A.B.C. Bilirubin enhanced spontaneous firings of MVN neurons by primarily increasing the amplitude of I_inward_, and the augment still existed even after 20-min washing. 0.2 mM ascorbate acid was added in perfusion to avoid oxidation of bilirubin except for base section. **p < 0.01, ***p < 0.001, NS, not significant, one-way ANOVA with LSD *post hoc* test for frequency comparison and Tamhane's T2 test for spike amplitude analysis.
